# Metabolic Effects of Violet Light on Spoilage Bacteria from Fresh-Cut Pakchoi during Postharvest Stage

**DOI:** 10.3390/plants11030267

**Published:** 2022-01-19

**Authors:** Yuchen Zhang, Zhaoyang Ding, Jing Xie

**Affiliations:** 1 College of Food Science and Technology, Shanghai Ocean University, Shanghai 201306, China; d200300070@st.shou.edu.cn (Y.Z.); zyding@shou.edu.cn (Z.D.); 2 National Experimental Teaching Demonstration Center for Food Science and Engineering, Shanghai Ocean University, Shanghai 201306, China; 3 Shanghai Professional Technology Service Platform on Cold Chain Equipment Performance and Energy Saving Evaluation, Shanghai 201306, China

**Keywords:** 405 nm light-emitting diodes, metabolic changes, *Pseudomonas*, ultrahigh-performance liquid chromatography (UHPLC)

## Abstract

Pakchoi (*Brassica rapa* L. *Chinensis*) is an important vegetable in Asia. *Pseudomonas palleroniana* is one of the specific spoilage organisms (SSOs) of fresh-cut pakchoi. The purpose of this study was to investigate changes to the endogenous metabolic spectrum of violet light (405 nm) with regard to food spoilage bacteria from fresh-cut pakchoi using ultrahigh-performance liquid chromatography-tandem mass spectrometry. In this study, *P. palleroniana* samples were treated with violet light at 4 °C, and the maximum dose was 133.63 J/cm^2^. The results revealed that 153 metabolites and 83 pathways significantly changed compared to the control group, which indicated that light treatment may lead to ROS accumulation in cells, inducing oxidative stress and the excessive consumption of ATP. However, the increased content of aromatic amino acids and the decreased anabolism of some amino acids and nucleotides might be a form of self-protection by reducing energy consumption, thus contributing to the improvement of the tolerance of cells to illumination. These results provide new insights into the antibacterial mechanism of *P. palleroniana* with regard to metabolism.

## 1. Introduction

Pakchoi (*Brassica rapa* L. *Chinensis*) is an important cruciferous vegetable in Asia. Fresh-cut vegetables refer to vegetables that have been washed, classified, peeled, or sliced. The tissues of these fresh-cut vegetables are rich in nutrients and have an almost neutral pH, which presents virtually ideal conditions for the survival and growth of many types of spoilage microorganisms [1]. *Pseudomonas* spp., one of the main spoilage bacteria related to fresh-cut vegetables, shows a high correlation with vegetable deterioration [2,3,4,5,6]. The results of our previous study showed that *Pseudomonas palleroniana* is one of the specific spoilage organisms (SSOs) of fresh-cut pakchoi, which causes pakchoi to decline in quality during the storage stage [7]. Nonetheless, previous studies revealed that LED_405 nm_ light effectively reduced bacterial levels on the surface of fresh-cut fruits or vegetables during the storage stage [8,9] and extended their shelf life [10]. For instance, Kim et al. (2017) discovered that LED_405 nm_ illumination significantly reduced populations of *Salmonella* on fresh-cut papaya by 0.3–1.3 log CFU/cm^2^ at 4 °C for 36–48 h (1.3–1.7 kJ/cm^2^, *p* < 0.05) [11]. Meanwhile, our previous study showed that LED_405 nm_ light can not only inhibit the level of spoilage bacteria on fresh-cut pakchoi during the storage stage but also prolong the shelf life of fresh-cut pakchoi [10]. With regard to the mechanism of the bacteriostatic method of visible light, several scholars believe that reactive oxygen species (ROS) are generated inside cells and destroy the bacterial structure, resulting in a sublethal state or even cell death, which is called photodynamic inactivation (PDI) [12,13,14]. PDI usually occurs when blue light (400–430 nm), oxygen, and photosensitizers are presented at the same time. However, there is no satisfactory correlation between microbial sensitivity and its intracellular photosensitizer level [15]. Therefore, it is necessary to investigate other factors, including other inactivation pathways and microbial responses to light treatment, which may play a key role in determining the degree of light inactivation with the help of metabolomics.

Metabolomics, which is an analytical method of studying all the metabolites in biological systems, reveals the functional state of organisms under different environmental conditions by detecting and analyzing intracellular metabolites [16]. Recently, metabolomics has been applied in disease research [17], food health research [18], plant and animal research [19], microorganisms [20,21], and other fields as a powerful and valuable emerging tool. Huan Zhang et al. [22] found significant changes in amino acid, sugar, and lipid contents of *Pediococcus pentosaceus* under oxidative stress after the addition of H_2_O_2_ via a metabolomic approach. Using metabolomics, Jorge Pamplona Pagnos et al. [23] found that natural bacteriostatic agents (NBAs) in the forms of nanoemulsions had significant sublethal effects on *Bacillus cereus*. Nonetheless, few studies have explained the bacteriostasis mechanism of 405 nm light from the perspective of metabolomics. Furthermore, UHPLC-MS/MS technology, which is sensitive, reliable, and powerful, can be used to study food spoilage bacteria.

Hence, this study aimed to investigate metabolic changes with regard to food spoilage bacteria (*P. palleroniana*) in fresh-cut pakchoi treated (PT) or not treated (PCK) with LED_405 nm_ light illumination at 4 °C via UHPLC-MS/MS; this approach attempted to provide new insights into the antibacterial mechanism of 405 nm light on food spoilage bacteria (*P. palleroniana*).

## 2. Materials and Methods

### 2.1. Culture of the Bacterial Strains

In our previous work, *Pseudomonas palleroniana* CFBP 4389 samples were isolated from spoiled pakchoi and were identified by 16S rRNA gene sequencing using a VITEK^®^2 CompactA system (BIOMÉRIEUX, France) [10]. Bacterial stock cultures contained 25% glycerine and were stored at −80 °C. The accession number of NCBI of *Pseudomonas palleroniana* CFBP 4389 is NR_029050.1. The culture of the bacterial strains was detailed by Wang et al. [24]. After incubation to a concentration of 10^8^ CFU/mL, the bacteria were centrifuged and resuspended using PBS solution, and this was repeated twice. The resuspended liquid was diluted to 10^5^ CFU/mL and then divided into the light-treated group (PT) and the control group (PCK). The PT group was treated with 405 nm light to the dose of 133.63 J/cm^2^ at 4 °C, while the PCK group was placed in the dark at 4 °C for the same amount of time. Each treatment was performed using 6 biological replicates.

### 2.2. Metabolite Extraction and Quality Control Sample

The methods of metabolite extraction were in accordance with the methods outlined by Wang et al. [24] with some modification. Samples (amounts of 100 μL) were added to 80 μL of methyl alcohol and 320 μL of water to extract metabolites with 0.02 mg/mL L-2-chlorophenylalanin as an internal standard. As a part of the system conditioning and quality control process, a pooled quality control sample (QC) was prepared by mixing equal volumes of all samples. The QC samples were disposed of and tested in the same manner as the analytic samples. This process helped to represent the whole sample set, which would be injected at regular intervals in order to monitor the stability of the analysis. The processing and testing methods used for the QC samples were the same as those of the analytical samples.

### 2.3. Data Preprocessing and Annotation

Mass spectra of metabolic features were identified by using the accurate mass, MS/MS fragments spectra, and isotope ratio differences, which were determined with searches in reliable biochemical databases such as the Human Metabolome Database (HMDB) and the Metlin database.

### 2.4. UHPLC-MS Analysis

Chromatographic separation of the metabolites was performed using a Thermo UHPLC system equipped with anshagn ACQUITY UPLC HSS T3. (100 × 2.1 mm i.d., 1.8 µm; Waters, Milford, CT, USA).

The mobile phases consisted of 0.1% formic acid in water: acetonitrile (95:5, *v*/*v*) (solvent A) and 0.1% formic acid in acetonitrile: isopropanol: water (47.5:47.5:5, *v*/*v*) (solvent B). The solvent gradient changed according to the following conditions: from 0 to 0.1 min, 0% B to 5% B; from 0.1 to 2 min, 5% B to 25% B; from 2 to 9 min, 25% B to 100% B; from 9 to 13 min, 100% B to 100% B; from 13 to 13.1 min, 100% B to 0% B; from 13.1 to 16 min, 0% B to 0% B to equilibrate the systems. The sample injection volume was 2 µL, and the flow rate was set to 0.4 mL/min. The column temperature was maintained at 40 °C. During the period of analysis, all these samples were stored at 4 °C.

The mass spectrometric data were collected using a Thermo UHPLC-Q Exactive Mass Spectrometer equipped with an electrospray ionization (ESI) source operating. Data acquisition was performed using the Data-Dependent Acquisition (DDA) mode. The detection was carried out over a mass range of 70–1050 m/z.

### 2.5. Multivariate Statistical Analysis

Principal component analysis (PCA) and orthogonal partial least squares discriminant (PLS-DA) were used with the ropls (Version 1.6.2, http://bioconductor.org/packages/release/bioc/html/ropls.html, accessed on 1 April 2021). R package from Bioconductor on the Majorbio Cloud Platform (http://cloud.majorbio.com, accessed on 1 April 2021). Variable importance in the projection (VIP) values was calculated using the OPLS-DA model. P-values were estimated with paired Student’s *t*-test in single-dimensional statistical analysis. Correlation analysis was performed using Pearson correlation test coefficient, and *p*-value < 0.05 was considered significant between PT vs. PCK.

### 2.6. Differential Metabolites Analysis

Differential metabolites among the two groups (PT and PCK) were summarized and mapped into their biochemical pathways through metabolic enrichment and pathway analysis based on the database search (KEGG, http://www.genome.jp/kegg/, accessed on 15 April 2021). Stats (Python package) (https://doce.scipy.org/doc/scipy/, accessed on 15 April 2021) was exploited to identify a statistically significantly enriched pathway using Fisher’s exact test.

## 3. Results and Discussion

### 3.1. Metabolic Variations among Light-Treated (PT) and Untreated (PCK) P. palleroniana

**Figure 1:**
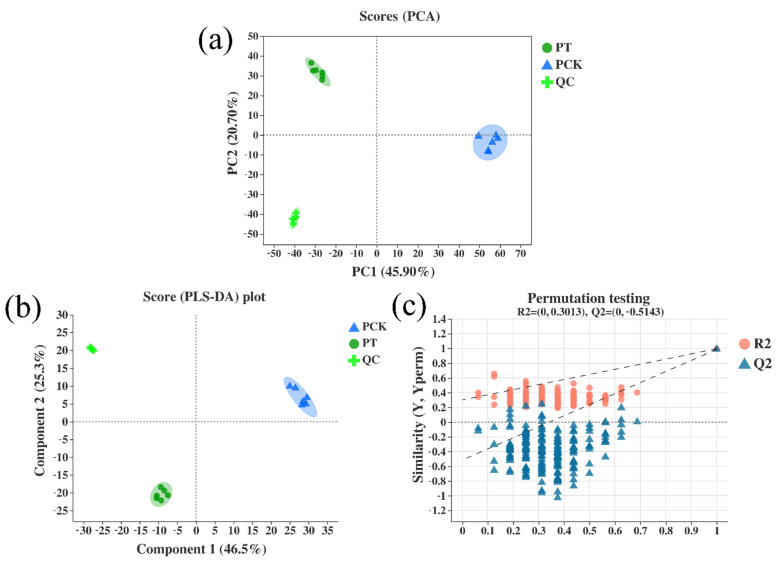
PCA score plots of treated and untreated *P. palleroniana* samples and QC samples (**a**). Validation of PLS-DA models of pairwise comparison among treated and untreated *P. palleroniana* samples (**b**,**c**). Abbreviations: quality control, QC; untreated *P. palleroniana*, PCK; light-treated *P. palleroniana*, PT.

The PCA score plot was responsible for 66.6% (PC1 45.9%, PC2 20.7%) of the overall variance of metabolite profiles, explaining a difference in the distribution of the PT and PCK groups. In a PCA score graph, the distance between the relative coordinate points of P1 and P2 represents the degree of aggregation and dispersion among the samples. The closer the distance is, the higher the similarity between the samples is. PCA analysis can be used to observe the trend of inter-group separation in the experimental model, and it can also determine whether there are outlier points and reflect the inter-group and intra-group variability from the original data. The confidence ellipse indicates that a set of “true” samples are distributed in a region with 95% confidence. In this study, the PCA score graph was constructed with P1 (R^2^X (cum) = 0.459) and P2 (R^2^X (cum) = 0.666), indicating that this model has suitable fitness ability. As can be seen in Figure 1a, the PCA analysis results showed that the contribution rate of PC1 was 45.9%, and that of PC2 was 20.7%; the PCK and PT groups were basically within the 95% confidence interval, and the detection results of the six repeated data points were basically well concentrated. In addition, QC validation is the basis for obtaining reliable and high-quality metabolomics data when conducting metabolomics studies based on mass spectrometry [25]. Figure 1a,b represent the PCA score diagrams of QC samples. The test results of red QC samples are clustered closely together, indicating that the repeatability is suitable.

We established the PLS-DA model scoring diagram (Figure 1b,c) of the control group (PCK) and the treatment group (PT). The data show that the fit good (R^2^X) and predictability (Q^2^) of the PT group and the PCK group were 0.492 and 0.520 (P1) and 0.788 and 0.997 (P2), respectively. Furthermore, permutation test cross-validation was performed 200 times to ensure the suitability of the model, and the fit good of the PT group and the PCK group R^2^X (cum), R^2^Y (cum), and Q^2^ (cum) were 0.492, 0.532, and 0.52 (P1) and 0.788, 0.997, and 0.997 (P2), respectively. The results show that there is no overfitting or false positives in the experimental data.

### 3.2. Correlation Analysis of Metabolites in Untreated P. palleroniana (PCK Group) and Treated P. palleroniana (PT Group)

**Figure 2:**
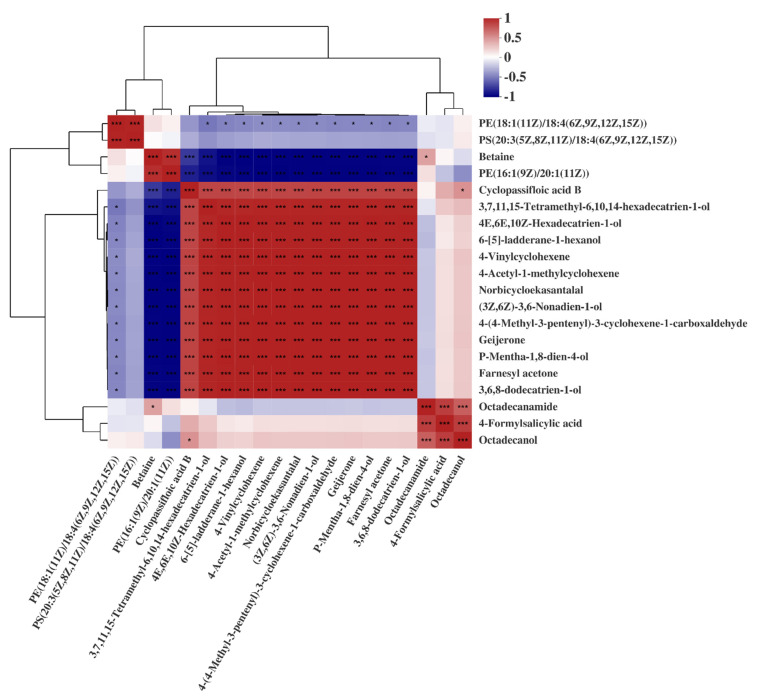
Pearson correlation analysis of treated and untreated *P. palleroniana*. Levels of significance are defined as * *p* < 0.05, and *** *p* < 0.001.

The correlation of the PCK and PT metabolites was studied via Pearson correlation analysis. Using each compound as a variable, the detailed results (top 20) are shown in Figure 2. The red region (Pearson correlation coefficient 0~1) indicates a positive correlation between the two substances, while the blue region indicates the opposite (Pearson correlation coefficient 0~−1). In the comparison of light-treated (PT) and untreated (PCK) *P. palleroniana*, PE (18:1(11Z)/18:4(6Z,9Z,12Z,15Z)) had no correlation with betaine, PE (16:1(9Z)/20:1(11Z)), cyclopassifloic acid B, octadecanamide, 4-formylsalicylic acid, and octadecanol. Furthermore, except for PE (18:1(11Z)/18:4 (6Z,9Z,12Z,15Z)) and PS (20:3(5Z,8Z,11Z)/18:4 (6Z,9Z,12Z,15Z)), PS (20:3(5Z,8Z,11Z)/18:4 (6Z,9Z,12Z,15Z)) was shown to have no correlation with other compounds in the top 20 (*p* < 0.05).

### 3.3. Qualitative and Quantitative Analysis of 405 nm Light-Treated and Untreated P. palleroniana

**Figure 3:**
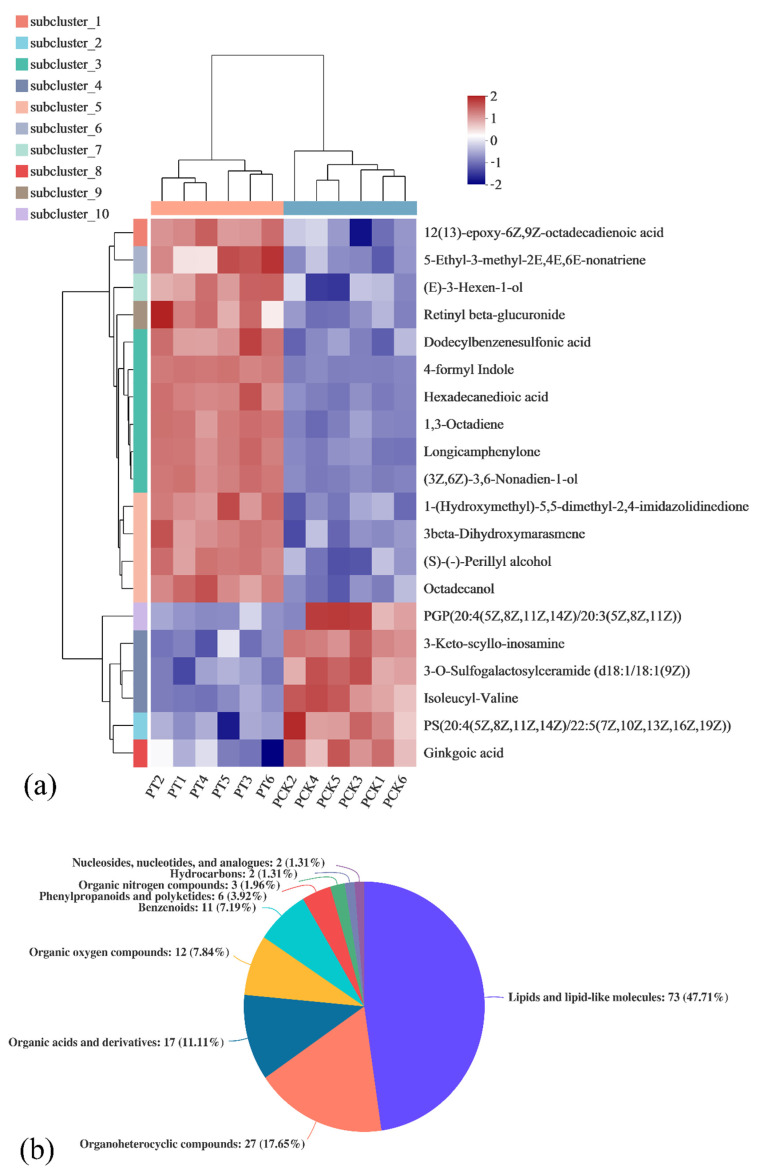
Comparison of the relative abundance of metabolites in PT (PT1, PT2, PT3, PT4, PT5, and PT6) vs. PCK (PCK1, PCK2, PCK3, PCK4, PCK5, and PCK6) groups (**a**), relative abundance of metabolites of light-treated and untreated *P. palleroniana* (**b**).

After data processing and analysis, we found that 153 metabolites presented metabolic differences (*p* < 0.05) between the PT and PCK groups, which include 73 lipids and lipid-like molecules, 27 organoheterocyclic compounds, 17 organic acids and derivatives, 12 organic oxygen compounds, 11 benzenoids, 6 phenylpropanoids and polyketides, 3 organic nitrogen compounds, 2 hydrocarbons, and 2 nucleosides, nucleotides and analogs. To study the metabolite correlation after light illumination in more detail, the samples of the PT group and the PCK group were compared. The relative abundance of metabolites of light-treated and untreated *P. palleroniana* (Figure 3a) and the expression profile and VIP of the top 30 metabolites (Figure 3b) are shown here. Statistically significant differences among groups were determined by VIP values of more than 1 and *p*-values less than 0.05.

Figure 3a shows the changes in the chemical compositions of cells after 405 nm light treatment in the form of a heat map. It has been proved that 405 nm light treatment can destroy cell membranes or other components [26,27]. In this study, it was observed that lipid substances in the PT group changed dramatically after treatment compared with the PCK group (Figure 3a,b). These results provide further support for this view and confirm that 405 nm light can significantly affect the metabolism of lipid substances in cells. Therefore, 405 nm illumination may affect the morphology of *P. palleroniana* by affecting the main substances in a cell membrane, such as phospholipids, and thus destroy its structure.

**Figure 4:**
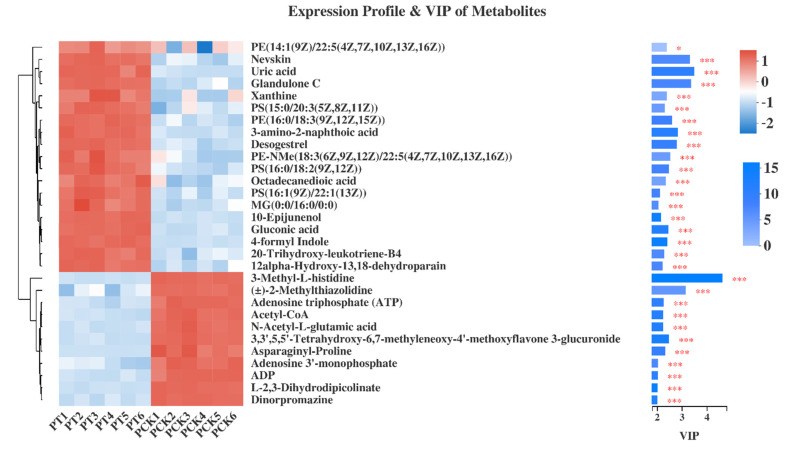
Expression profile and VIP of top 30 metabolites in PT (PT1, PT2, PT3, PT4, PT5, and PT6) vs. PCK (PCK1, PCK2, PCK3, PCK4, PCK5, and PCK6) group. Levels of significance are defined as * *p* < 0.05, and *** *p* < 0.001.

Figure 4 shows the expression profile and VIP value of the top 30 metabolites. Here, it is also shown that the abundance of lipids and lipid-like molecules significantly changed after being treated with light illumination (VIP > 2, *p* < 0.05), such as PE (14: 1(9Z) / 22: 5 (4Z, 7Z, 10Z, 13Z, 16Z)), glandulone, PS (15: 0 / 20: 3 (5Z, 8Z, 11Z)), PE (16: 0 / 18: 3 (9Z, 12Z, 15Z)), desogestrel, PE -NMe (18: 3 (6Z, 9Z, 12Z) / 22: 5 (4Z, 7Z, 10Z, 13Z, 16Z)), PS (16: 0 / 18: 2 (9Z, 12Z)), octadecanedioic acid, PS(16: 1(9Z) / 22: 1(13Z)), octadecanedioic acid, PS (16: 1(9Z) / 22: 1(13Z)), MG(0: 0 / 16: 0 / 0: 0), 10-epijunenol, 20-trihydroxy-leukotriene-B4, 12alpha-hydroxy-13, 18-dehydroparain and acetyl-CoA. At the same time, ATP (VIP = 2.2533, *p* < 0.001), ADP (VIP = 2.0131, *p* < 0.001) and acetyl-CoA (VIP = 2.2273, *p* < 0.001) were significantly down-regulated, indicating that the oxidative phosphoric acid metabolic pathway was down-regulated. In addition, it has previously been shown that the ratio of saturated to unsaturated fatty acids in bacterial cell membranes changes under peroxide or low-temperature stress [28]. In this experiment, it is possible that the change in lipid content was due to the production of ROS inside the bacteria by light, which caused oxidative stress to the bacteria.

### 3.4. Metabolic Pathway Analysis Based on the KEGG Database

**Figure 5:**
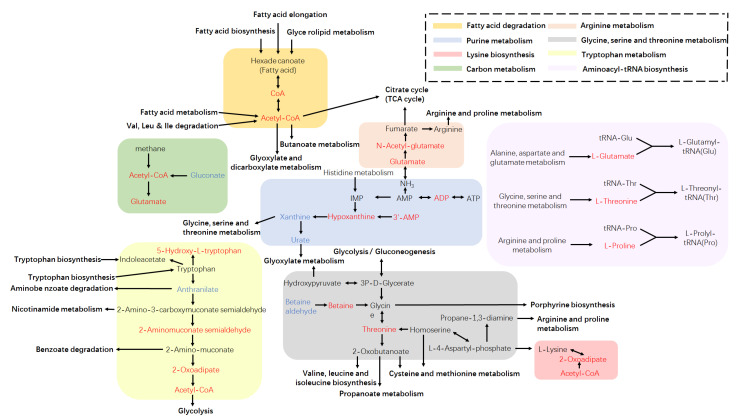
Changes in intracellular metabolites mapped to metabolic pathways for PT and PCK groups. The metabolites marked in blue and red indicate significant increases and decreases, respectively, in PT compared with PCK groups (*p* < 0.05).

The metabolic pathways were identified according to the KEGG database to determine the most evident and vital metabolic or biosynthetic pathways related to the metabolites. A total of 83 metabolic pathways were significantly changed after light treatment, which was annotated by the KEGG database. Changes in intracellular metabolites mapped to the metabolic pathways for the PT and PCK groups are shown in Figure 5.

**Figure 6:**
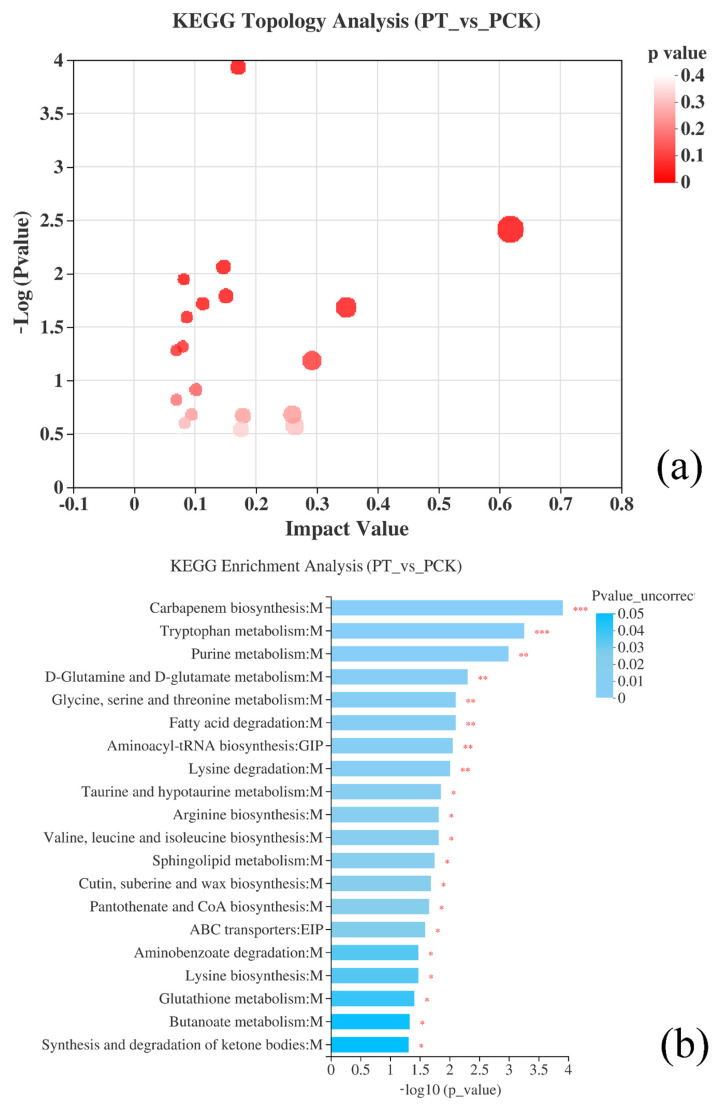
KEGG topology analysis (PT vs. PCK) (**a**), the 20 most enriched ratios of pathway terms of KEGG enrichment in PT vs. PCK groups (**b**). Levels of significance are defined as * *p* < 0.05, ** *p* < 0.01, and *** *p* < 0.001.

Recently, studies revealed that PDI occurred through ROS generation inside cells and destroying the bacterial structure through a type I or type II mechanism, when blue light, porphyrins, and oxygen were present at the same time. However, there is no satisfactory correlation between microbial sensitivity and the porphyrin level [15]. Therefore, it is necessary to explore these mechanisms in depth with the help of metabolomics. In this study, the KEGG-enriched metabolic analysis results showed the top 10 different metabolic pathways (Figure 6), including amino acid pathways (tryptophan metabolism; D-glutamine and D-glutamate metabolism; glycine, serine, and threonine metabolism; aminoacyl–tRNA biosynthesis; lysine degradation; arginine biosynthesis; valine, leucine and isoleucine biosynthesis; and lysine biosynthesis), the energy supply pathway (pantothenate and CoA biosynthesis), the purine pathway (purine metabolism), the antioxidant pathway (glutathione metabolism), and other pathways (carbapenem biosynthesis; fatty acid degradation; taurine and hypotaurine metabolism; sphingolipid metabolism; cutin, suberine, and wax biosynthesis; ABC transporters; aminobenzoate degradation; butanoate metabolism; and the synthesis and degradation of ketone bodies).

Amino acids are major metabolites in the biosynthesis and metabolism of many cells, where they are usually involved in cellular construction as precursors and regulate cellular metabolism through the formation of catalase. Thus, changes in some amino acids’ content are indicative of oxidative stress in cells [29]. Here, the results show that differential metabolites involved in the amino acid metabolism containing L-glutamate, L-threonine, L-proline, 3-methyl-L-histidine, acetyl-CoA L-isoleucine, and L-methionine were significantly down-regulated. In contrast, 2-aminobenzoic acid, L-tryptophan, and glutathione were significantly up-regulated (Figure 6).

**Figure 7:**
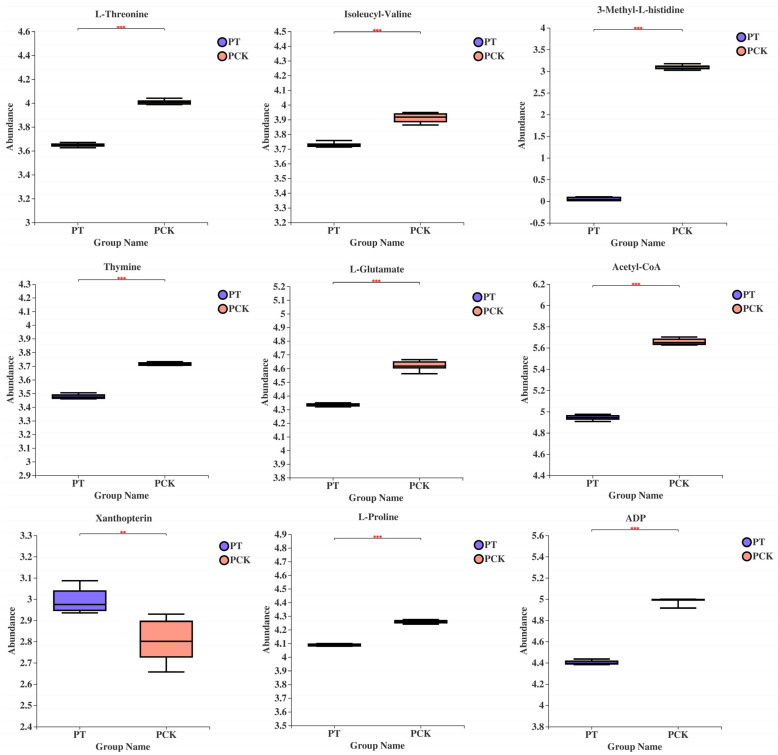
Box plot of some significantly different metabolite content levels in *P. palleroniana* treated (PT) or not treated (PCK) with 405 nm light illumination.

Metabolomic analysis showed a significant decrease in intracellular L-glutamate, L-threonine, L-proline, 3–methyl-L-histidine, and acetyl-CoA after 405 nm light irradiation (Figure 7). Previous studies have revealed that cells synthesize new protective proteins to combat environmental changes under oxidative stress [30,31], and L-glutamate can act as an ROS scavenger against oxidative stress [32]. In the present study, the oxidized glutathione content decreased significantly under the influence of 405 nm light irradiation. This could be due to the fact that 405 nm light LED accumulated ROS in cells, which disturbed the redox balance of cells and generated oxidative stress, and *P. palleroniana* cells consumed a large amount of L-glutamate to maintain normal cell growth to counteract the damage caused by ROS. Therefore, it is speculated that cells provide energy and reduce the power to resist light-induced oxidative stress through L-glutamate catabolism, which leads to a decrease in L-glutamate content.

The stress response of microorganisms requires an energy supply, so the conservation of energy is particularly important [33]. Jozefczuk et al. [31] found that environmental stresses such as low-temperature stress lead to a decrease in the content of substances related to the carbon center metabolism, which is a method of self-protection used by cells to reduce energy consumption under resistance to stress. In this experiment, both acetyl-CoA and glutamate levels in the carbon metabolism were significantly reduced after light treatment, indicating that the cells reduced their energy supply in order to conserve energy. In addition, the content of ADP and ATP in the cells was significantly reduced (Figure 6), which is also a form of self-protection achieved by energy conservation measures taken by *P. palleroniana* cells. However, the lack of energy supply to the cells resulted in a significant depletion of acetyl-CoA after light treatment (*p* < 0.001) and its inability to be replenished, leading to the involvement of acetyl-CoA in valine, leucine and isoleucine degradation, valine, leucine, and isoleucine biosynthesis, lysine biosynthesis and lysine degradation, and arginine and proline metabolism, histidine metabolism, phenylalanine metabolism, and beta-alanine metabolism were significantly reduced after 405 nm light treatment. This suggests that the reduced energy metabolism triggered a chain reaction that resulted in blocked protein synthesis, making *P. palleroniana* unable to synthesize proteins to compensate for the broken protein structure and inhibiting the ability to maintain normal life activities.

The L-amino acid content for tRNA ligation is generally decreased in the aminoacyl–tRNA biosynthetic pathway, which may be closely related to the regulation of cell growth and metabolism because of the crucial role of the aminoacyl–tRNA synthesis pathway in protein synthesis. Weber et al. [34] found that under environmental stress conditions, genes related to cell growth, division, and protein synthesis were repressed during environmental stress to reduce energy loss and achieve self-protection. Thus, we speculate that 405 nm light can cause nutrient amino acid deprivation in *P. palleroniana* cells, resulting in insufficient aminoacyl–tRNA biosynthesis substrates, blocked protein biosynthesis, and the inhibition of cell growth, while *P. palleroniana* cells reduce energy requirements by reducing amino acid metabolic pathways, maintaining carbon and nitrogen metabolic balance and allowing them to survive under adverse conditions.

Nucleotides are essential substances for cell growth and reproduction, which not only serve as precursors for DNA and RNA synthesis but also play important roles in metabolism and can be involved in the metabolism of substances in the body as regulatory substances for physiological and biochemical processes [35]. There is a close connection between nucleotide metabolism and amino acid metabolism, such as the shared metabolites of 5-phosphoribosyl pyrophosphate, 5-aminoimidazole-4-carboxamide nucleotide, tetrahydrofolate, etc. LED_405 nm_ light affects amino acid metabolism, which inevitably also affects intracellular nucleotide metabolism. Metabolomic analysis shows that metabolites related to the purine metabolism in *P. palleroniana* cells were severely inhibited under the stress of LED _405 nm_ light, and their levels were shown to generally be lower than those in PCK groups. The contents of ADP, hypoxanthin, and adenosine 3’-monophosphate were significantly down-regulated, while the metabolism of uric acid and xanthine were significantly up-regulated, as shown in Figure 6.

Purines and pyrimidines are important components of intracellular nucleotide metabolism, while most nucleotide synthesis is an energy-consuming process [36]. However, in the purine metabolism, we found that the content of several metabolites, such as hypoxanthine and adenosine 3’-monophosphate, decreased significantly. In addition, there was a decrease in thymine content, which is involved in pyrimidine metabolism, and the synthesis of these metabolites is also energy intensive. In addition, the oxidative phosphorylation metabolism was significantly down-regulated (*p* < 0.05), while the presence of ROS caused impaired intracellular ATP production and increased consumption. It is evident that *P. palleroniana* cells choose to reduce the biosynthesis of nucleotide metabolism and decrease energy consumption to improve tolerance to ROS. In addition, the levels of metabolites involved in the nucleoside synthesis pathway were generally down-regulated, and pyrimidine metabolism was significantly affected (*p* < 0.05), suggesting that 405 nm inhibits DNA biosynthesis in *P. palleroniana* cells, which in turn affects normal cell growth. It has been well documented that 405 nm light causes elevated levels of bacterial DNA oxidation [37], whereas the results of the present experiment found that the decreased expression of functional metabolism such as oxidative phosphorylation leads to impaired function and ultimately to impaired repair of bacterial DNA synthesis, providing a deeper explanation for such ideas.

Previous studies have revealed that enzymes involved in the steroid metabolism produce ROS by leaking electrons to NADPH in the lack of matrix, resulting in cell death [38]. If the purine metabolism is indeed affected, it indicates that fluctuations in some metabolic pathways that do not involve porphyrins may also contribute to bacterial photoinactivation. Purines are essential as precursors of nucleotides, signal molecules (such as cAMP and GMP), and central energy currency ATP that make up DNA and RNA [39]. Meanwhile, metabolites related to purine metabolism, including ADP, 3’AMP, hypoxanthine, xanthine, and urate, were also greatly affected during light treatment. Furthermore, some metabolites produced by biosynthetic pathways were influenced by the light illumination, such as the tryptophan metabolism and carbapenem biosynthesis, which changed significantly with acetyl-CoA, 2-Oxoadipate, 2-Aminomuconate semialdehyde, anthranilate, 5-Hydroxy-L-tryptophan and L-proline, L-glutamate, CoA, and pantetheine.

These results suggest that photoinactivation may be guided not only by endogenous porphyrins in bacterial cells but also by other molecules and pathways, including amino acids pathways, energy supply pathways, purines, and pyrimidines pathways, antioxidant pathways, etc. It is worth noting that this study is a proof of concept and did not take place in a real food system. Therefore, in an actual food system, the results of this study should be carefully inferred, and the actual inactivation level may vary depending on the type of food.

## 4. Conclusions

UHPLC-MS-based non-targeted metabolomics revealed 153 metabolites and 83 pathways with significantly differential amounts in LED_405 nm_ light-treated and untreated samples of *P. palleroniana*. The analysis of differential metabolic pathways showed that 405 nm light treatment may lead to ROS accumulation in cells and induce oxidative stress and the excessive consumption of ATP, which are the main reasons for inhibiting the growth of cells. However, the increased content of aromatic amino acids and the decreased anabolism of some amino acids and nucleotides may be a form of self-protection by reducing energy consumption, thus contributing to the improvement of the tolerance of cells to illumination. These results provide new insights into the antibacterial mechanism of *P. palleroniana* with regard to metabolism.

## Data Availability

The data presented in this study are available on request from the corresponding author.
